# Examining the Efficacy of Five Lactobacillus Species in Treating and Preventing Atopic Dermatitis: A Systemic Literature Review

**DOI:** 10.7759/cureus.64833

**Published:** 2024-07-18

**Authors:** Imina Emokpae, Deanna L Tobia, Saskia D Stamm, Petra Lundy, Derek S Weimer, Michelle Demory Beckler

**Affiliations:** 1 Medical School, Nova Southeastern University Dr. Kiran C. Patel College of Allopathic Medicine, Fort Lauderdale, USA; 2 Biomedical Sciences, Nova Southeastern University Dr. Kiran C. Patel College of Allopathic Medicine, Fort Lauderdale, USA; 3 Family and Community Medicine, Nova Southeastern University Dr. Kiran C. Patel College of Allopathic Medicine, Fort Lauderdale, USA; 4 Microbiology and Immunology, Nova Southeastern University Dr. Kiran C. Patel College of Allopathic Medicine, Fort Lauderdale, USA

**Keywords:** medicine, microbiome, probiotics, eczema, atopic dermatitis, lactobacillus, microbiology, nutrition, dermatology, nutrition and dermatology

## Abstract

Probiotics have garnered increasing attention, particularly within the realm of atopic dermatitis (AD). Although classified as dietary supplements by the Food and Drug Administration, probiotics are being explored for their potential to modify immune system responses and aid in disease recovery. This review aims to provide a current understanding of probiotics, specifically various lactobacilli strains, as a therapeutic option in preventing and treating AD.

The concept of the gut-skin axis has gained substantial recognition, emphasizing the complex relationship between the gut microbiome and skin health. Dysfunctional gut barriers and metabolites produced by gut microorganisms can exert profound influences on skin conditions, including AD. Lactobacilli species are particularly noteworthy for their resilience and stability within the gastrointestinal tract, making these bacteria ideal candidates for probiotic supplementation. Various lactobacilli strains (*Lactobacillus salivarius*,* Lactobacillus acidophilus*,* Lactobacillus plantarum*,* Lactobacillus reuteri*,* and Lactobacillus rhamnosus*) were included in this study due to their current uses in mitigating AD symptomatology.

This systemic review article aims to shed light on the potential of probiotics as a therapeutic approach for AD, highlighting their stellar safety profile and promising therapeutic efficacy. Given the compelling preliminary findings and the constraints associated with conventional treatments, probiotics, particularly lactobacilli strains, emerge as a considerable alternative or adjuvant option for individuals grappling with AD. Further exploration is imperative to establish probiotics as a promising therapeutic option, providing renewed hope for those seeking effective strategies for managing AD.

## Introduction and background

Atopic dermatitis (AD) is clinically characterized as a highly pruritic, chronic inflammatory disease with an underlying pathophysiology that is complex and not completely understood. AD is considered the most common inflammatory skin disorder in children and has a prevalence of approximately 10% in adults [1]. Though common in children and adults, infants are the most affected population [2]. Patients with AD commonly present with flexural accentuation of an erythematous rash that can appear violaceous in patients with darker skin and is oftentimes pruritic [3]. AD poses significant social, emotional, and economic burdens with a large negative influence on the quality of life of those affected.

In addition to a complex pathophysiology, the risk associated with AD is multifactorial. The interplay between genetic susceptibility, environmental factors, and immune dysregulation appears to be chiefly responsible for the clinical manifestations. One of the most well-studied genetic mutations lies within the *FLG* gene responsible for the production of the filaggrin protein, an integral component of maintaining a healthy epidermis [4]. With mutations documented in approximately 20-25% of AD patients, the filaggrin protein is instrumental in skin barrier integrity. A mutation within this gene and subsequent protein products can lead to disruption throughout the stratum corneum, the most superficial layer of the epidermis, predisposing the skin to an increased frequency of infections [5,6].

According to the American Academy of Dermatology, the first-line treatment options for patients with AD include adhering to a routine skincare regimen, managing triggers, and applying topical medications (e.g., topical corticosteroids, calcineurin inhibitors, and coal tar) [7]. Second-line options encompass light therapy and systemic medications, though efficacy is largely dependent on the severity of the disease and quality of life exhibited by the patient [8]. Newer therapies such as oral corticosteroids and injectable biological therapies (biologics) have demonstrated significant improvement in clinical symptoms for both children and adults with moderate-to-severe AD [9]. However, due to potential adverse effects, these are reserved as second-line agents [9]. Adverse effects of steroids include increased susceptibility to opportunistic infections (secondary to immunosuppression), hypertension, glucose intolerance, and adrenal suppression [9]. Additionally, discontinuation of steroid use often leads to rebound flares, prompting recommendations for short-term use primarily in refractory cases or as transitional therapy [9]. Several biologics have received United States Food and Drug Administration (FDA) approval for moderate-to-severe AD with the first being approved in 2017 [8,10]. Biologic therapy targets specific interleukins (ILs) implicated in AD pathogenesis, potentially minimizing side effects while maximizing efficacy [8]. Despite their promise, severe adverse reactions may necessitate alternative therapies, either as adjuvants or monotherapy [11].

Probiotics have a long history of safe use spanning over a century, with rare and limited side effects. Adverse effects, though uncommon, may include bacteremia and minor gastrointestinal (GI) symptoms such as abdominal cramping, nausea, and taste disturbance [12,13]. Contraindications primarily apply to immunocompromised individuals due to potential bacterial influence on immune system functioning [14]. Common bacteria found in probiotic supplements include lactobacilli, *Bifidobacterium*, lactococci, and various yeasts [14]. Deemed generally regarded as safe by the FDA, probiotics demonstrate minimal risk in children and adult populations when comparing safety profiles to conventional therapies [14]. Unlike steroid use, probiotics are not limited by duration of use. While efficacy varies compared to conventional therapies, the risk of severe and adverse side effects remains minimal, suggesting potential as an additional therapeutic approach for AD.

*Lactobacillus*, a lactic acid bacteria (LAB) species, natively lives in the human intestine and exerts health benefits to the host through various properties [15]. LAB, including *Lactobacillus*, have demonstrated the production of antimicrobial substances, antioxidants, antimutagens, prevention of pathogen colonization, and regulation of genes associated with inflammation [16]. Additionally, *Lactobacillus* exhibits optimal survivability through the GI tract, making it an ideal candidate for use in probiotic supplementation [16]. When assessing efficacy in AD, strain-specific research is particularly crucial due to significant variability across different *Lactobacillus* strains.

Although nutraceutical options have been available for many years, probiotic supplements have only recently become an area of interest in preventing and treating AD [17]. Due to their immunomodulatory effects in oral formulations, probiotics have been shown to be a suitable addition or alternative to conventional medical regimens [17]. However, research studies on probiotic efficacy, particularly at the strain-specific level, remain limited. This review aims to provide a current understanding of *Lactobacillus* as a therapeutic option for preventing and treating AD.

## Review

Atopic dermatitis pathogenesis and biologic treatments

Immune system dysregulation is a prominent feature in the pathogenesis of AD. Accordingly, hyperimmune function can lead to degradation of the epidermal barrier defense, contributing to a decreased protective role of the skin microbiome and surrounding tissues [18]. The breakdown of the skin barrier allows allergens and pathogens to penetrate, exacerbating the inflammatory cycle [18]. The subsequent pathogenic irritation results in the activation of naive CD4+ T cells, leading to downstream production of specific CD4+ helper T (Th) cell subsets, including Th1, Th2, Th17, Th22, and regulatory T cells (Treg) cells [18]. The pro-inflammatory cytokines and chemokines produced from these cells can create a positive feedback loop leading to magnified immune responses characteristic of AD pathogenesis.

Effector CD4+ T cells and their pro-inflammatory cytokines play pivotal roles in both the initiation and progression of AD. Specific cytokines contribute to epithelial barrier dysfunction, overactive immune responses, reduction in skin antimicrobial peptides, and elevated serum IgE levels, all hallmark features of AD [18]. These immune alterations lead to skin inflammation and pruritus, common in those with AD lesions [18]. Notably, non-lesional skin manifestations in adults with chronic AD also express similar T-cell activation and cytokine profiles to lesional skin, underscoring the systemic nature of AD [19].

The imbalance of Th1/Th2 cytokines in AD has been extensively studied. Acute AD is characterized by early Th2 dominance, while chronic disease presents with Th1 dominance [20].

Specific cytokines, such as IL-4, IL-13, IL-22, and IL-31, show increased expression in acute AD, while cytokines derived from Th1 and Th17 cells exhibit smaller increases in expression [20]. Th2 cytokines such as IL-4 and IL-13 suppress the expression of epidermal barrier genes, exacerbating epithelial barrier defects in AD [21]. Additionally, IL-5, a Th2 cytokine, promotes the maturation of eosinophils, B cells, and secretion of IgE, thereby intensifying allergic responses and contributing to AD progression [21]. Consequently, Th2 cytokines have become successful drug targets in AD therapy.

Novel biologic therapies targeting specific Th2 mediators have been developed for managing AD. Dupilumab, an anti-IL-4 biologic, for example, has led to significant improvements in the SCORAD index (a tool to asses AD severity) and a reduction in total IgE serum levels [22]. Additionally, lebrikizumab and tralokinumab, anti-IL-13 biologics, have been shown to resolve AD symptoms in patients with moderate-to-severe AD [23]. Studies involving biologics have not only improved the management of AD but also demonstrated the importance of targeting specifically Th2 dysregulation and associated cytokines that drive AD pathogenesis.

Contrary to Th2 cells, Th1 cells produce cytokines such as interferon-gamma (IFN-𝛾) and IL-2 which are responsible for immune activation and inflammation [24]. Though IL-4 and IL-13, Th2 cytokines, have been shown to suppress IFN-𝛾-induced human 𝛽-defensin-3 (HBD-3), an antimicrobial peptide produced in epithelial cells, IFN-𝛾 itself plays a role in skin hypertrophy [24,25]. Recombinant subcutaneous IFN-𝛾 (rIFN-𝛾) injections have shown promise in moderate-to-severe AD [26], though only indicated in a small population of pediatric patients with a distinct phenotype who are prone to skin infections such as herpes simplex virus and *Staphylococcus aureus*, thus limiting its use [26]. Few biologics target solely Th1 activity, likely due to the minimal involvement of Th1-mediated reactions in AD.

In chronic AD, Th17 cells are elevated in peripheral blood and lesions of AD patients, releasing cytokines critical in inflammation and skin barrier dysfunction [27]. Specifically, IL-17, a Th17 cytokine, decreases the expression of filaggrin. Additionally, increased expression of IL-8 can be identified, causing recruitment of immune cells, increased vascular endothelial growth factor with subsequent increases in vascular permeability, and increased levels of CXCL10, causing dysregulation in both innate and adaptive immune responses [27]. These cytokine characteristics contribute to the inflammatory response observed in chronic AD when Th1 is elevated [27]. The increased downstream effects produced by these cytokines further suggest a role of Th17 in the inflammatory process and skin barrier breakdown characteristic of AD. IL-22, another cytokine produced by Th17 cells, has been associated with epidermal hyperplasia and decreased keratinocyte differentiation in AD [19]. Targeting IL-22 with the biologic, fezakinumab, showed an average reduction of 13.8 SCORAD (severity scoring of atopic dermatitis) scores in patients with severe AD, marking great clinical improvement [19].

In addition to the key role of Th2 and Th1 cells in AD, some studies have implicated Treg. Treg cell activation, marked by Forkhead box-p3 (*FOXP3*) gene expression, is decreased in the serum of AD patients. Treg cells induce apoptosis of antigen-presenting cells and regulate the activity of T cells and their consequent pro-inflammatory and allergic responses [28]. Given these functions, reductions in Treg cells may contribute to further unregulated immune activation in AD. A study using AD murine models found that decreased FOXP3+ expression, the transcription factor that indicates Treg activity, was correlated to an increased inflammatory response. In the same study, Treg cells were shown to regulate levels of IL-4, IL-10, and IL-13 as well as IgE production and eosinophil activation [28]. These findings suggest that Tregs play a role in AD by attenuating Th2 activity and highlight the role of decreased Treg cell regulation in the pathogenesis of AD.

Atopic dermatitis and probiotics

The underlying pathophysiology of AD has prompted the development of various biologic treatments, though concerns about adverse reactions, uncertain long-term implications, and cost have led to an exploration of alternative therapies. Probiotic therapy, which influences gut microbial diversity, has emerged as one such option.

Research into the gut microbiome’s influence on skin has recently intensified. Studies highlight the critical role of gut microbial diversity in regulating overall immunity and disease risk throughout life [29]. Probiotics, supplements that modulate gut microbial composition, have shown promise in preventing and treating various diseases, including antibiotic-associated diarrhea, *Helicobacter pylori* infections, and irritable bowel syndrome, though AD has not yet been greatly considered [30].

*Lactobacillus*, a major component of probiotic supplements, is a genus within the group considered as LAB with over 170 species that natively live in the human intestine and exert health benefits to the host [15]. LAB are known for their production of antimicrobial substances, antioxidants, and antimutagens, as well as their ability to prevent pathogen colonization and regulate inflammation-associated genes [16]. With their survivability and stability in the GI tract, along with their ability to adhere to the intestinal wall, lactobacilli are appropriate candidates for use in probiotic supplements [16]. Notably, strain-specific research is crucial due to unique variations in efficacy among bacterial strains.

Below, we provide an overview of major probiotic lactobacilli strains investigated for their potential role in AD management.

Lactobacillus salivarius

*Lactobacillus salivarius* (*L. salivarius*) has demonstrated various anti-inflammatory properties providing therapeutic benefits in various conditions, including asthma, cancer, and GI infections specifically related to the ingestion of swine and poultry [17]. In the context of AD, *L. salivarius* is primarily administered orally, although other routes of administration have also been explored [17].

Among the various strains of *L. salivarius*, LSO1 has been shown to significantly decrease the severity of AD in both adults and children. A study involving adults with AD found that supplementation with LSO1, in combination with *Streptococcus thermophilus* ST10, significantly reduced SCORAD and improved Dermatological Life Quality Index scores compared to LSO1 alone [31]. These beneficial effects were attributed to the ability of LSO1 to trigger the production of Treg cells with a subsequent influence on the Th1/Th2 cytokine ratio in individuals with AD [31,32].

Children have also shown improvement with oral *L. salivarius* treatment. In one study, *L. salivarius* LSO1 strain treatment significantly reduced SCORAD and itch index scores in children aged newborn to 11 years after four weeks of treatment [33]. Another study involving 60 children aged 2 to 14 years showed that treatment with *L. salivarius* PM-A000 strain and prebiotic fructooligosaccharides led to a significant reduction in SCORAD scores when compared to fructooligosaccharide treatment alone [34]. The same study also found that serum eosinophilic cationic protein levels were significantly reduced with treatment [34].

In a mouse model, the *L. salivarius* LA307 strain demonstrated efficacy in reducing skin inflammation by significantly decreasing pro-inflammatory cytokines IL-17 and IL-22, while increasing anti-inflammatory cytokine IL-10 levels [35]. These findings further suggest the immunomodulatory role of *L. salivarius* in the treatment of AD.

Overall, various strains of *L. salivarius* have shown promise in alleviating AD symptoms. While previous research credits various underlying immunological mechanisms in reducing AD severity, there is a commonality among these attributed to the reduction of IgE activation [33-35]. However, due to the diversity of *L. salivarius* strains and the common practice of combining them with other prebiotics and probiotics, an individualized stepwise mechanism poses a challenge. Further research focusing on individual strains of *L. salivarius* is warranted to better understand their efficacy and mechanisms of action in AD management.

Lactobacillus acidophilus

*Lactobacillus acidophilus *(*L. acidophilus*) is commonly found in various bodily microenvironments such as the vagina, intestines, and oral cavity. Oral formulations have shown promising effects in reducing allergy-related symptoms, particularly in the L-92 strain [36-38]. Studies investigating the immunoregulatory properties of *L. acidophilus* L-92 have yielded encouraging results, particularly in the context of AD treatment [36-38].

In a preliminary double-blind, placebo-controlled study, the complementary effects of long-term oral treatment of L-92 were evaluated in pediatric patients with AD [39]. The study showed statistically significant time-dependent changes in the symptom score of Atopic Dermatitis Area and Severity Index. Furthermore, reductions in medication scores (i.e., a numerical value designated based on the amount of conventional therapy used) were observed along with a decline in pruritis (p <0.001) and overall AD manifestations. Notably, significant decreases were observed in total white blood cell counts with decreasing concentrations of eosinophils throughout the duration of the study [39]. These findings suggest a comprehensive alleviation of AD symptoms with L-92 supplementation in pediatric populations.

While research in pediatric populations has shown promise, research in adult populations remains limited. One double-blind study consisting of 49 adolescent and adult patients studied the L-92 strain in those with AD. Administered orally, L-92 was implemented as an adjuvant therapy to the patient’s conventional treatment plan, including corticosteroid ointment, oral histamines, and moisturizers [40]. The results indicated significant reductions in SCORAD indexes and decreased eosinophil counts in those treated with L-92 compared to controls [40]. Although the exact anti-inflammatory mechanism of L-92 remains unclear, it is speculated to influence intracellular transduction and the production of cytokines such as TGF-𝛽, IL-8, and prostaglandins [41].

While these findings are promising, limitations such as the concurrent use of other medications and lack of standardization within treatment groups highlight the need for further research to elucidate the immune mechanisms underlying the effectiveness of *L. acidophilus*, particularly in adult patients.

Lactobacillus plantarum

*Lactobacillus plantarum* (*L. plantarum*) is a naturally occurring gut bacteria known for its anti-inflammatory and immunomodulatory properties, making it a potentially valuable candidate for treating pro-inflammatory conditions such as AD [42].

As an immune-modulator, *L. plantarum* has been found to improve AD symptoms in various studies. In a randomized, double-blind study involving pediatric patients, oral supplementation with *L. plantarum* CJLP133 led to significantly decreased SCORAD scores in the treatment group after 14 weeks [43]. These improvements were associated with significant reductions in IL-4 and IFN-𝛾 serum levels, indicating modulatory effects on Th1 and Th2 cell activity [43]. Similarly, 22 children with mild or moderate AD and high IgE levels were treated with *L. plantarum* IS-10506 twice daily for 12 weeks [44]. Treatment resulted in significant decreases in SCORAD scores and serum IL-4, IL-17, and IFN-𝛾 concentrations. Significant increases were noted in the Treg cell transcription factor FOXP3 as well as serum IL-10 [44]. Collectively, these studies suggest that supplementation with *L. plantarum* provides a shift in the Th1/Th2 ratio toward homeostasis, ultimately leading to improved clinical symptoms.

In vivo studies have further elucidated the underlying mechanism of action of *L. plantarum* in AD. In a mouse model demonstrating contact dermatitis, administration of *L. plantarum* and/or 𝛽-1,3/1,6-glucan (fungus-derived soluble prebiotic) was conducted for seven days. T-cell analysis revealed significantly increased expression of Th1 and Treg transcription factor genes, T-bet, and FOXP3 [45]. Expression of Th2 and Th17 transcription factor genes, *GATA*-3 and *RORγT*, in addition to their cytokines, IL-4 and IL-17, respectively, showed significant reductions compared to controls [45]. These findings suggest that *L. plantarum* can modulate the Th1/Th2 and Treg/Th17 ratios in a way that corrects immune imbalance and reduces inflammation in AD.

Additionally, the same study revealed reductions in thymic stromal lymphopoietin (additional marker in AD-associated inflammation) and histamine along with increased levels of Galectin-9 and filaggrin, further highlighting the potential of *L. plantarum* in mitigating various aspects of AD pathogenesis.

While these studies demonstrate the promising effects of *L. plantarum* in AD treatment, further research is needed to understand its efficacy in differentiating between acute (Th2 dominant) and chronic (Th1 dominant) AD and to elucidate its specific mechanisms of action.

Lactobacillus reuteri

*Lactobacillus reuteri* (*L. reuteri*) is a prevalent colonization species in the GI tract of various mammals. Its beneficial effects on inflammatory disease modulation are attributed to the production of potent antimicrobial metabolites such as 3-hydroxypropionaldehyde (3-HPA), also known as reuterin. Produced during the metabolism of glycerol, reuterin is effective in inhibiting the growth of gram-positive and gram-negative bacteria, yeasts, protozoa, and fungi [46]. Although the production of 3-HPA has been observed in other bacterial species, *L. reuteri* uniquely produces excess amounts of 3-HPA greater than bioenergetically required, contributing to its probiotic efficacy [47].

Research examining the use of *L. reuteri* in AD often focuses on prophylactic probiotic supplementation in infants and children. While studies show promise in reducing AD flares, supplementation with these bacteria does not appear to completely prevent the onset of AD. In one double-blind randomized placebo-based trial, 188 pregnant women with a history of allergic disease were started on either probiotic or placebo supplementation for four weeks before term with continuation until delivery. After birth, the baby continued taking the same supplementation as the mother for 12 months, with follow-up at 1, 3, 6, 12, and 24 months of age. It was found that while the cumulative incidence of AD was similar in the probiotic and placebo groups, the incidence of IgE-associated AD (measured via skin prick test) was decreased in the probiotic group [48]. These findings suggest potential long-lasting benefits in the infant from short-term *L. reuteri* supplementation during pregnancy.

Interestingly, through antimicrobial and modulatory effects on the skin barrier junction, *L. reuteri* has been shown to be more effective in treating AD when applied topically rather than orally. A recent clinical study including 36 adults with AD demonstrated the cutaneous acceptability and tolerability of topically applied *L. reuteri* [49]. Significant improvements in SCORAD scores, as well as overall itching and dryness, were observed when applied topically in adults with AD [49]. These findings highlight the unique topical applicability of *L. reuteri*.

Lactobacillus rhamnosus

*Lactobacillus rhamnosus* (*L. rhamnosus*) is well-known for its ability to regulate GI health, facilitated by its unique capability to adhere to the intestinal mucosa through surface pili, prolonging exposure within the GI tract [50]. Consequently, it is commonly used as a probiotic supplement and is also integrated into many dairy products such as cheese and yogurt [51]. Despite extensive research on its GI benefits, its effects on AD remain uncertain. A systematic review and meta-analysis concluded that evidence supporting the supplementation of *L. rhamnosus* for reducing the risk of developing AD is lacking [52].

One study investigated the combined effects of *L. reuteri *and *L. rhamnosus* supplementation in children with AD. This double-blind, placebo-controlled, crossover study involving children aged 1 to 13 years observed the clinical effects of either dual probiotic or placebo supplementation in alleviating AD [53]. The group receiving active treatment reported a 56% improvement in AD incidence compared to the placebo group, which showed a 15% improvement (p = 0.001) [53]. Although the SCORAD index decreased after administration of *L. reuteri* and *L. rhamnosus*, the reduction was not statistically significant [53]. The inconclusive evidence may be attributed to the inclusion of two different strains. Overall, there appears to be no known significant benefit of *L. rhamnosus* supplementation as a standalone therapy for AD. However, large, well-controlled studies are needed to further explore its possible benefits.

Discussion

Probiotics are living microorganisms that convey health benefits to the host, including the potential to manage AD [12]. These microorganisms, resilient enough to withstand the acidic pH of the stomach and reach the lower intestinal tract epithelium, exert various effects that contribute to human health [54]. Their abilities include outcompeting pathogenic microorganisms, enhancing intestinal barrier function, synthesizing critical nutrients, and maintaining and regulating immune system functions [54-56].

While considered dietary supplements by the FDA, probiotics, particularly those that include *Lactobacillus* strains, have shown promise in modifying immune responses and aiding in disease recovery [30,57,58]. However, their potential remains largely unproven due to a lack of robust scientific studies in addition to prevalent false claims in the supplement market [59]. Though lacking FDA approval, probiotics are commonly used to improve conditions causing gut dysbiosis [30]. Ongoing research into various body systems influenced by the gut microbiome raises the question of whether probiotics can serve as a therapeutic option for non-enteric disorders, such as skin pathologies like AD.

In the realm of AD management, *L. salivarius*, *L. acidophilus*, *L. plantarum*, and *L. reuteri* have demonstrated efficacy in alleviating AD symptoms, with *L. salivarius* and *L. plantarum* being the most well-studied [15]. These strains exert beneficial effects on AD pathophysiology through immune cell modulation, cytokine regulation, and enhancement of gut barrier integrity, showing promise in reducing SCORAD indices, decreasing serum cytokine levels, and increasing the abundance of Treg cells [15,55].

The concept of the gut-skin axis highlights the bidirectional communication between the gut microbiome and the skin. In healthy tissue, the tight epithelial layer guarding the intestinal tract selectively prevents the absorption of foreign antigens into the systemic circulation. In addition, gut-associated lymphoid tissue plays a vital role in the differentiation of Th cells, offering additional protective support [60]. Conversely, individuals with gut dysbiosis exhibit compromised epithelial layers, enabling the absorption of immunogenic antigens, with the subsequent development of various systemic inflammatory reactions [61]. This idea has been applied to various inflammatory skin conditions in which immune hyperactivity is the underlying driver [56]. Although the exact physiological mechanisms underlying the gut-skin connection remain incompletely understood, these findings emphasize the importance of maintaining a healthy gut microbiome. Further understanding of the gut-skin axis may elucidate how oral probiotic formulations, including lactobacilli species, exert their effects on the skin.

In clinical practice, probiotics offer a promising alternative or adjunct to conventional AD treatments. With their favorable safety profile, probiotics may provide additional benefits and fewer adverse effects compared to traditional therapies [7]. However, further research is needed to elucidate the specific mechanisms underlying the efficacy of probiotics in AD management, identify optimal strains and dosages, and establish their role as complementary therapies for AD [30]. Large-scale, well-controlled studies are warranted to validate the potential of probiotics in enhancing the quality of life for individuals affected by AD.

## Conclusions

AD is a multifactorial inflammatory skin disorder with a complex pathophysiology and a significant impact on patients’ quality of life. While conventional treatments exist, including topical medications and biologic therapies, their efficacy may be masked due to adverse effects. Probiotics, particularly lactobacilli strains, have emerged as promising adjunctive or alternative therapies for AD management. *Lactobacillus* species exert beneficial effects on AD pathophysiology through immune modulation, cytokine regulation, and enhancement of gut barrier integrity. Various strains, including *L. salivarius*, *L. acidophilus*, *L. plantarum*, *L. reuteri*, and *L. rhamnosus*, have demonstrated efficacy in alleviating AD symptoms, showing promise in reducing SCORAD indices, decreasing serum cytokine levels, and increasing the abundance of Treg cells. The concept of the gut-skin axis highlights the bidirectional communication between the gut microbiome and the skin, underscoring the potential of oral probiotic formulations to influence cutaneous health. While probiotics offer a favorable safety profile and may provide additional benefits compared to traditional therapies, further research is needed to elucidate their specific mechanisms of action, identify optimal strains and dosages, and establish their role as complementary therapies for AD.
